# Supplementary material for: Impacts of COVID-19 Restrictions on Young Children’s Outdoor Activity: A Systematic Review
Source: Children (Basel). 2022 Oct 16;9(10):1564. doi: 10.3390/children9101564 (PMC9600871; doi:10.3390/children9101564)
Supplement: Supplementary file 1 [file children-09-01564-s001.zip › Supplementary File S1.pdf]

Supplementary File S1-Search strategies

1. Education Research Complete, Psychology and Behavioral Sciences Collection, SPORTDiscus with Full Text (EBSCOhost)

Search date: 22/11/2021

(COVID-19 or coronavirus or "COVID-19 pandemic" or 2019-ncov or sars-cov-2 or cov-19 or pandemic or lockdown or "social distanc*" or "School clos*") AND (outdoor or "outdoor spaces" or outside or nature or park or playground or garden or "front yard" or "green space*" or neighborhood or street*) AND (play or "free play" or "unstructured play" or "independent mobilit*" or activit* or "unorganized physical activit*" or "organized physical activit*") AND (child* or kid or boy or girl or infant or toddler or "early years" or youth)

Results: 130 articles (No limits)

Limited to peer-reviewed : 109; limited after 2020: 95; limited to English: 94

2. ERIC (ProQuest)

Search date: 22/11/2021

(COVID-19 or coronavirus or "COVID-19 pandemic" or 2019-ncov or sars-cov-2 or cov-19 or pandemic or lockdown or "social distanc*" or "School clos*") AND (outdoor or "outdoor spaces" or outside or nature or park or playground or garden or "front yard" or "green space*" or neighborhood or street*) AND (play or "free play" or "unstructured play" or "independent mobilit*" or activit* or "unorganized physical activit*" or "organized physical activit*") AND (child* or kid or boy or girl or infant or toddler or "early years" or youth)

Results: 44 articles (No limits)

Limited to peer-reviewed: 21; limited after 2020: 15; limited to English: 13

3. WHO COVID-19 Database

Search date: 22/11/2021

Title, abstract, subject: COVID-19 and outdoor activity and child

Results: 26 articles (No limits)

Limited to English: 25; limited after 2020: 25

4. Ovid MEDLINE(R) , APA PsycInfo

Table S1: Search date: 22/11/2021

#	Query	Results from 16 Nov 2021
1	(COVID-19 or coronavirus or 2019-ncov or sars-cov-2 or cov-19 or pandemic or lockdown).mp. [mp=ti, ab, ot, nm, hw, fx, kf, ox, px, rx, ui, an, sy, tc, id, tm, mh]	141,434
2	"COVID-19 pandemic".mp. [mp=ti, ab, ot, nm, hw, fx, kf, ox, px, rx, ui, an, sy, tc, id, tm, mh]	40,475
3	(social distanc* or school clos*).mp. [mp=ti, ab, ot, nm, hw, fx, kf, ox, px, rx, ui, an, sy, tc, id, tm, mh]	9,851
4	(outdoor or outside or nature or park or playground or garden or neighborhood).mp. [mp=ti, ab, ot, nm, hw, fx, kf, ox, px, rx, ui, an, sy, tc, id, tm, mh]	421,796
5	("outdoor spaces" or "front yard").mp. [mp=ti, ab, ot, nm, hw, fx, kf, ox, px, rx, ui, an, sy, tc, id, tm, mh]	230
6	(green space* or street*).mp. [mp=ti, ab, ot, nm, hw, fx, kf, ox, px, rx, ui, an, sy, tc, id, tm, mh]	20,317
7	play.mp. [mp=ti, ab, ot, nm, hw, fx, kf, ox, px, rx, ui, an, sy, tc, id, tm, mh]	297,574
8	("free play" or "unstructured play").mp. [mp=ti, ab, ot, nm, hw, fx, kf, ox, px, rx, ui, an, sy, tc, id, tm, mh]	3,479
9	(independent mobilit* or activit* or unorganized physical activit* or organized physical activit*).mp. [mp=ti, ab, ot, nm, hw, fx, kf, ox, px, rx, ui, an, sy, tc, id, tm, mh]	1,047,936
10	child*.mp. [mp=ti, ab, ot, nm, hw, fx, kf, ox, px, rx, ui, an, sy, tc, id, tm, mh]	1,214,649
11	(kid or boy or girl or infant or toddler or youth).mp. [mp=ti, ab, ot, nm, hw, fx, kf, ox, px, rx, ui, an, sy, tc, id, tm, mh]	409,511
12	"early years".mp. [mp=ti, ab, ot, nm, hw, fx, kf, ox, px, rx, ui, an, sy, tc, id, tm, mh]	5,850
13	1 or 2 or 3	146,562
14	4 or 5 or 6	438,951
15	7 or 8 or 9	1,293,392
16	10 or 11 or 12	1,382,755

17	13 and 14 and 15 and 16	149 (No limits)
18	limit 17 to english language	136
19	limit 18 to peer reviewed journal [Limit not valid in Ovid MEDLINE(R); records were retained]	127
20	limit 19 to yr="2020 -Current"	107
