# Supplementary material for: Impacts of COVID-19 Restrictions on Young Children’s Outdoor Activity: A Systematic Review
Source: Children (Basel). 2022 Oct 16;9(10):1564. doi: 10.3390/children9101564 (PMC9600871; doi:10.3390/children9101564)
Supplement: Supplementary file 1 [file children-09-01564-s001.zip › Supplementary File S2.pdf]

Supplementary Tile S2 Quality assessment

Table S2: Outcome of the critical appraisal of the included studies (Cross sectional studies)

S/No	Study reference	Criteria assessed based on JBI checklist								Total criteria met
		1	2	3	4	5	6	7	8	
1	Ma et al. 2021	Y	Y	Y	Y	Y	Y	Y	Y	8
2	Moore et al. 2020	Y	N	N	Y	Y	N	Y	Y	5
3	Dunton et al. 2020	Y	Y	Y	U	Y	Y	Y	Y	7
4	Moore et al. 2021	Y	N	Y	Y	Y	N	Y	Y	6
5	Schnaiderman et al. 2021	Y	Y	Y	N	N	N	Y	Y	5
6	Delisle Nystrom et al. 2020	Y	Y	Y	Y	Y	Y	Y	Y	8
7	Okely. et al. 2021	Y	Y	Y	Y	Y	Y	Y	Y	8
8	Nathan et al. 2021	Y	Y	Y	Y	Y	N	Y	Y	7
9	Yum et al. 2021	Y	Y	U	U	N	N	N	Y	3
10	Poulain et al. 2021	Y	Y	Y	N	Y	Y	U	Y	6
11	Shneor et al. 2021	Y	Y	Y	Y	N	N	Y	Y	6

Criteria 1-8: 1: clarity of inclusion criteria in the sample; 2: detailed description of the study subjects and settings; 3: measured exposure in a valid and reliable way; 4:objective, standard criteria for measurement of the condition; 5: identified confounding factors; 6:stated strategies to deal with confounding factors; 7: measured outcomes in a valid and reliable way; 8: appropriateness of statistical analysis. Key: Y=Yes, N=No, U=Unclear.

Table S2: Outcome of the critical appraisal of the included studies (Qualitative study)

S/No	Study reference	Criteria assessed based on JBI checklist										Total criteria met
		1	2	3	4	5	6	7	8	9	10	
1	Lafave, L.et al. 2021	Y	Y	Y	Y	Y	Y	Y	Y	Y	U	9
2	Pelletier et al. 2021	Y	Y	Y	Y	Y	N	Y	Y	Y	Y	9
3	Pascal et al. 2021	Y	Y	Y	Y	Y	Y	Y	Y	N	Y	9
4	Neshteruk et al. 2021	N	Y	Y	Y	Y	N	Y	Y	Y	Y	8

Criteria 1-10: 1: congruity between the stated philosophical perspective and the research methodology; 2: congruity between the research methodology and the research question or objectives; 3: congruity between the research methodology and the methods used to collect data; 4: congruity between the research methodology and the representation and analysis of data; 5: congruence between the research methodology and the interpretation of results; 6: locating the researcher culturally or theoretically; 7:

influence of the researcher on the research, and vice-versa, is addressed; 8: representation of participants and their voices; 9: ethical approval by an appropriate body; 10: relationship of conclusions to analysis, or interpretation of the data. Key: Y=Yes, N=No, U=Unclear.
